# The morphogenic protein CopD controls the spatio-temporal dynamics of PBP1a and PBP2b in *Streptococcus pneumoniae*

**DOI:** 10.1128/mbio.01411-23

**Published:** 2023-09-20

**Authors:** Cassandra Lenoir, Anaïs Pelletier, Sylvie Manuse, Hugo Millat, Adrien Ducret, Anne Galinier, Thierry Doan, Christophe Grangeasse

**Affiliations:** 1 Molecular Microbiology and Structural Biochemistry, UMR, Université de Lyon, CNRS, Lyon, France; 2 Laboratoire de Chimie Bactérienne, UMR, Aix-Marseille Université, CNRS, Marseille, France; 3 Laboratoire d’Ingénierie des Systèmes Macromoléculaires, UMR, Aix-Marseille Université, Marseille, France; Universite de Geneve, Geneva, Switzerland

**Keywords:** peptidoglycan, penicillin-binding proteins, cell division, cell morphogenesis, *Streptococcus pneumoniae*

## Abstract

Penicillin-binding proteins (PBPs) are key to the assembly of peptidoglycan, the major component of the bacterial cell wall. Although several PBP-specific regulatory proteins have been identified in different species, little is known about how the activity of PBPs is controlled and coordinated during the cell cycle. In this study, we characterize the unknown function protein Spr1400 and demonstrate its regulatory function on two PBPs in *Streptococcus pneumoniae*. For that, we use a combination of technics ranging from bacterial genetics and protein biochemistry to microscopy imaging. First, we show that pneumococcal Spr1400 localizes late to the cell division septum. Furthermore, deletion of *spr1400* results in wider cells. Using co-immunoprecipitation and bacterial two hybrid (B2H), we observe that Spr1400 interacts with two PBPs, the class A PBP PBP1a and the class B PBP PBP2b, which are required for cell elongation. Microscale thermophoresis combined with B2H further reveals that these interactions occur through their transmembrane domains. We also show that Spr1400 co-localizes with PBP1a and PBP2b throughout the cell cycle. Strikingly, deletion of *spr1400* alters the dynamics of PBP1a and PBP2b. Indeed, the two PBPs persist longer at the division site and localize later at the division site of daughter cells. Collectively, these data demonstrate that Spr1400, thus named CopD for coordinator of PBP1a and 2b dynamics, is a spatio-temporal regulator of PBP1a and PBP2b required for pneumococcal morphogenesis.

**IMPORTANCE:**

Penicillin-binding proteins (PBPs) are essential for proper bacterial cell division and morphogenesis. The genome of *Streptococcus pneumoniae* encodes for two class B PBPs (PBP2x and 2b), which are required for the assembly of the peptidoglycan framework and three class A PBPs (PBP1a, 1b and 2a), which remodel the peptidoglycan mesh during cell division. Therefore, their activities should be finely regulated in space and time to generate the pneumococcal ovoid cell shape. To date, two proteins, CozE and MacP, are known to regulate the function of PBP1a and PBP2a, respectively. In this study, we describe a novel regulator (CopD) that acts on both PBP1a and PBP2b. These findings provide valuable information for understanding bacterial cell division. Furthermore, knowing that ß-lactam antibiotic resistance often arises from PBP mutations, the characterization of such a regulator represents a promising opportunity to develop new strategies to resensitize resistant strains.

## INTRODUCTION

Most bacteria are surrounded by an extracellular cell wall whose composition varies from species to species (1). An essential and conserved component of the cell wall is the peptidoglycan (PG), which forms an intricate network of glycan strands cross-linked by short peptides, and ensures the shape and physical integrity of the cell (2, 3). PG assembly begins at the inner leaflet of the membrane with the synthesis of a building block (Lipid II) by the Mur proteins (4). Lipid II is composed of a disaccharide of GlcNac-MurNac and a pentapeptide containing D- and L-amino acids and whose composition varies among species. Lipid II is then flipped across the membrane by the flippase MurJ and polymerized into a giant cage-like polymer by two types of PG synthases, the penicillin-binding proteins (PBPs) and the shape, elongation, division, and sporulation (SEDS) proteins (4 – 6).

Glycan strand polymerization and cross-linking is enzymatically well characterized. By contrast, the assembly mechanisms of the PG three-dimensional structure are diverse and complex among bacteria, not only to accommodate their specific cell shape and growth mode but also to ensure cell integrity during the cell cycle (7, 8). For that, bacteria have evolved a diversity of strain-specific proteins, regulatory processes, and notably different SEDSs and PBPs to direct the assembly of PG at the division septum and/or the lateral side and/or the pole (4, 9). Classically, the glycosyltransferase (GT) activity of two SEDS homologs, FtsW and RodA, is involved in the polymerization of glycan strands required for cell division and elongation, respectively (10, 11). However, the production of the PG layer may require multiple SEDS, as in *Listeria monocytogenes,* which requires two FtsW and three RodA (12). This is also true for the PBPs as their number also varies from species to species (13). In addition, while all PBPs possess a transpeptidase (TP) activity that cross-links the glycan strands by peptide bridges, some also possess a TG activity allowing glycan strand polymerization. PBPs are thus classified in two distinct families, the class A (aPBPs) which possesses both TP/TG activities and the class B (bPBPs) which possesses only the TP activity (13). The current model proposes that bPBPs work together with the SEDS to build the PG primary framework whereas the aPBPs are required for PG remodeling to maturate the primary PG framework during cell growth (11, 14). To maturate the PG layer, the activity of PBPs should therefore be tightly coordinated in space and time. The characterization of LpoA, LpoB, CpoB, and FtsN has pioneered this aspect by demonstrating that these three proteins control the two class A PBP1a and PBP1b in *Escherichia coli* (15).

*Streptococus pneumoniae* (pneumococcus) is a Gram-positive bacterial pathogen responsible for several diseases such as otitis, meningitidis, and pneumonia and that is commonly found in patients with chronic obstructive pulmonary disease (COPD) (16, 17). It is also a major model to study bacterial cell division and morphogenesis (18 – 20). In *S. pneumoniae*, PG is assembled only at midcell by the cell division machinery (divisome) (19). It is proposed that the bPBP/SEDS pairs PBP2x/FtsW and PBP2b/RodA assemble two types of primary PG, the septal and the peripheral PG, which would be responsible for the synthesis of the cross-wall and cell elongation, respectively. Recently, this view has been challenged and it is proposed that the ovoid shape of the pneumococcus relies on the continuous insertion of peripheral PG inside the septal PG rather than two successive phases of peripheral and septal synthesis (21). In addition, another study proposes that the three aPBPs (PBP1a, 1b, and 2a) of the pneumococcus would function autonomously to either repair the defects of the primary PG or make it denser and stronger (22). While the function of PBP1b remains elusive and is only proposed to participate in peripheral PG synthesis, PBP1a and PBP2a are crucial for the assembly of a mature cell wall (23). Although not essential, their co-deletion is synthetically lethal (23). To date, only three proteins have been shown to affect the function of class A PBPs in *S. pneumoniae*. The membrane protein CozEa (originally named CozE) (24), which regulates the localization of PBP1a, the homologous protein CozEb which also probably interferes with the function of PBP1a but indirectly (25) and MacP, which is reported to be an activator of PBP2a (26).

Recently, the membrane protein TseB (for tetracycline sensitivity *s*uppressor of *ezrA*) (27) has also been proposed to interfere with the function of the bPBP PBP2A during cell elongation and spore germination in *Bacillus subtilis* (28). A homologous protein of unknown function Spr1400 is found in the pneumococcus but its function has never been investigated. In this report, we first show that the pneumococcus does not behave like *B. subtilis* with respect to tetracycline sensitivity, ruling out a TseB-like effect for Spr1400. We also show that Spr1400 localizes to the division septum and is required for proper cell morphogenesis. We further demonstrate that Spr1400 interacts not only with the bPBP PBP2b, the counterpart of PBP2A in *B. subtilis*, but also with the aPBP PBP1a. Localization experiments further demonstrate that Spr1400 is required for the spatio-temporal dynamics of these two PBPs during the cell cycle. We thus named Spr1400 CopD for coordinator of PBP1a and 2b dynamics. These results thus identify the first protein that affects the function of both aPBPs and bPBPs. They also agree well with the current model of pneumococcal PG assembly and pave the way toward our further understanding of the coordination between the synthesis of primary peripheral PG with its remodeling and repair. More generally, they illustrate the complexity of the network of protein interactions required for PG assembly.

## RESULTS

### Depletion of EzrA does not impact tetracycline sensitivity of *S. pneumoniae*

**Fig 1:**
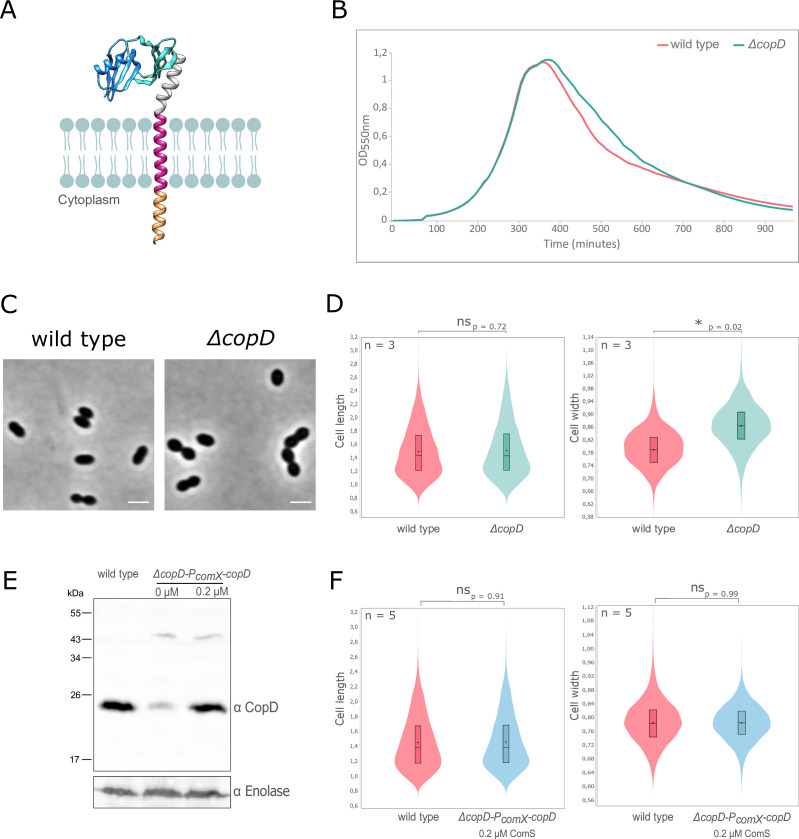
CopD and cell morphology and growth. (**A**) Schematic model for CopD. The two PEPSY domains were modeled using Alphafold (https://alphafold.com/entry/Q8DP25) and are adapted from published structural information of PEPSY-domains (PDB ID 5BOI). The predicted transmembrane domain and the short cytoplasmic domain (16 amino acids) are shown as red and orange α-helices, respectively. The two PEPSY domains are shown in dark and light blue. (**B**) Growth of WT and ∆*copD* strains. Strains were grown in C + Y medium at 37°C in a spectrophotometer. The OD_550_ was read automatically every 10 minutes. (**C**) Representative phase contrast microscopy images of WT and ∆*copD* cells. Scale bar, 2 µm. (**D**) Violin plot showing the distribution of the cell length (left panel) and cell width (right panel) for WT and ∆*copD* strains as determined using MicrobeJ (31). The distribution of the cell length and width are shown in red for the WT strain and in blue for the ∆*copD* strain. Statistical comparison was done using *t*-test. *P < 0.05. *n* = 3 indicates the number of independent experiments with a total of 5,000 cells analyzed. (**E**) Western immunoblot of whole-cell lysates from WT and ∆*copD-P_comX_-copD* cells, grown to exponential phase in the presence (0.2 µM) or absence of the ComS inducer, were probed with anti-CopD antibody. To estimate the relative quantity of proteins in crude extract and to compare the different lanes, we used the enolase (Spr1036) as an internal standard. The enolase was detected using specific antibodies (α Enolase) and is presented in the lower part of the Figure. (**F**) Violin plot showing the distribution of the cell width for WT and ∆*copD-P_comX_-copD* strains as determined using MicrobeJ (31). The distribution of the cell length (left panel) and cell width (right panel) is shown in red for the WT strain and in blue for the ∆*copD-P_comX_-copD* strain. Statistical comparison was done using *t*-test. *n* = 5 indicates the number of independent experiments with a total of 10,000 cells analyzed. For panels **D** and **F**, the box indicates the 25th to the 75th percentile, and the whiskers indicate the minimum and the maximum values. The mean and the median are indicated with a dot and a line in the box, respectively.

CopD is a membrane protein homologous to TseB from *Bacillus subtilis* (24.26% sequence identity and 33.73% similarity) sharing the same organization with two PEPSY domains (28) in its extracellular domain (Fig. 1A). Deletion of *tseB* has been reported to suppress the hypersensitivity to tetracycline of an *ezrA*-deficient *B. subtilis* strain (27). To determine if the same was true for *S. pneumoniae*, we first determined a sublethal concentration of tetracycline for the WT strain. In the presence of 15 ng/mL tetracycline, an intermediate growth rate was detected, whereas in the presence of 1.5 ng/mL tetracycline or 150 ng/mL tetracycline, growth was almost unaffected or completely abolished, respectively (Fig. S1A). Since *ezrA* is essential in the pneumococcus (29, 30), we constructed an *ezrA*-depletion strain (∆*ezrA-P_comX_-ezrA). ezrA* expression increased with ComS concentration, with a similar amount of EzrA produced as WT cells with 2 µM ComS (Fig. S1B). In this condition, this mutant strain grew like the WT strain, whereas it is unable to grow in the absence of ComS (Fig. S1C). Importantly, for this experiment, *ezrA* was under-expressed in presence of 1 µM ComS and cells grew poorly (Fig. S1B and C). We, therefore, used 1 µM ComS to analyze the effect of 1.5 and 15 ng/mL tetracycline on the growth of the ∆*ezrA-P_comX_-ezrA* strain. As shown in Fig. S1D, the presence of tetracycline did not further alter the growth when *ezrA* was depleted. We also analyzed cell viability and found no differences (Fig. S1E). Taken together, these experiments showed that the depletion of *ezrA* did not affect tetracycline sensitivity. Therefore, *S. pneumoniae* does not behave like *B. subtilis* with respect to tetracycline sensitivity (27), making further investigation about the association between *copD* and *ezrA* irrelevant for this phenotype.

### CopD is required for cell morphogenesis and localizes at mid-cell

To investigate the role of CopD in the pneumococcus, we constructed a markerless deletion mutant. ∆*copD* and WT showed similar growth profiles (Fig. 1B). Under the microscope, deletion of *copD* did not significantly affect the ovoid shape of pneumococcal cells (Fig. 1C). However, morphometric measurements of cells clearly showed that ∆*copD* cells are significantly wider than WT cells, while the cell length was not affected (Fig. 1D). Indeed, the mean cell width for WT cells was 0.79 ± 0.03 µm whereas it was 0.87 ± 0.04 µm for ∆*copD* cells. This observation was accompanied by a decrease in the population of cells with smaller width (4% of ∆*copD* cells have a width below 0.75 µm compared to 25% of WT cells) and an increase in the population of cells with larger width (74% of ∆*copD* cells have a width above 0.83 µm compared to 25% of WT cells). To exclude a downstream polar effect on the other genes of the same chromosomal locus, we constructed a complementation strain in which we introduced an ectopic copy of *copD* under the control of the inducible *comX* promoter in the ∆*copD* strain (∆*copD-P_comX_-copD*). Using polyclonal antibodies specific to CopD, we observed that *copD* was expressed at a similar level than in WT cells after induction with 0.2 µM ComS (Fig. 1E and F). Likewise, a WT phenotype was restored with a mean cell width of 0.79 µm ± 0.02 and the same distribution of cell width in the population of ∆*copD* cells than in WT cells (21% of ∆*copD* cells have a width below 0.75 µm compared to 25% of WT cells and 21% of ∆*copD* cells have a width above 0.83 µm compared to 25% of WT cells) (Fig. 1F). These results suggest that CopD plays a role in cell morphogenesis.

**Fig 2:**
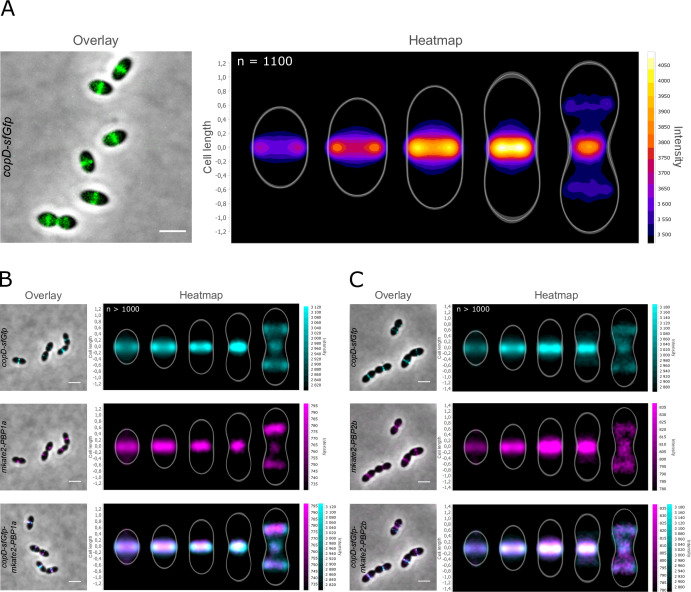
Localization of CopD-sfGFP, mKate2-PBP1a, and mkate2-PBP2b. (**A**) Overlays (left panel) between phase-contrast and GFP images of CopD-sfGFP cells. Scale bar, 2 µm. The heatmap (right panel) represents the localization patterns of CopD-sfGFP during the cell cycle. (**B**) and (**C**) co-localization of CopD-sfGFP and either mkate2-PBP1a (**B**) or mkate2-PBP2b (**C**) in WT cells. Overlays between phase contrast and GFP and mKate2 images are shown on the left while corresponding heatmaps representing the two-dimensional localization patterns during the cell cycle are shown on the right. Scale bar, 2 µm. The *n* values represent the number of cells analyzed in a single representative experiment. Experiments were performed in triplicate.

To determine the localization of CopD, we constructed a C-terminal sfGFP fusion to CopD (strain *copD-*sf*Gfp*). Throughout this study and unless otherwise indicated, protein fusions were constructed at each native chromosomal locus and expressed under the control of the native promoter, and represented the only source of protein. The CopD-sfGFP fusion was stable (Fig. S2A) and appeared to be fully functional as the cells grew as WT cells (Fig. S2B) and showed no significant change in cell width (Fig. S2C). As shown in Fig. 2A, CopD-sfGFP localized exclusively at the division septum. More precisely, the analysis of its dynamics in the course of the cell cycle showed that CopD-sfGFP is present at the division septum throughout the cell cycle. Interestingly, it re-localized to the cell equator, which corresponds to the future division site of the daughter cells, only at the very late stage of the cell cycle (Fig. 2A). Collectively, these data indicate that CopD is a cell division protein that is likely required for proper morphogenesis of the pneumococcus throughout the cell cycle.

### CopD interacts and co-localizes with PBP1a and PBP2b

**Fig 3:**
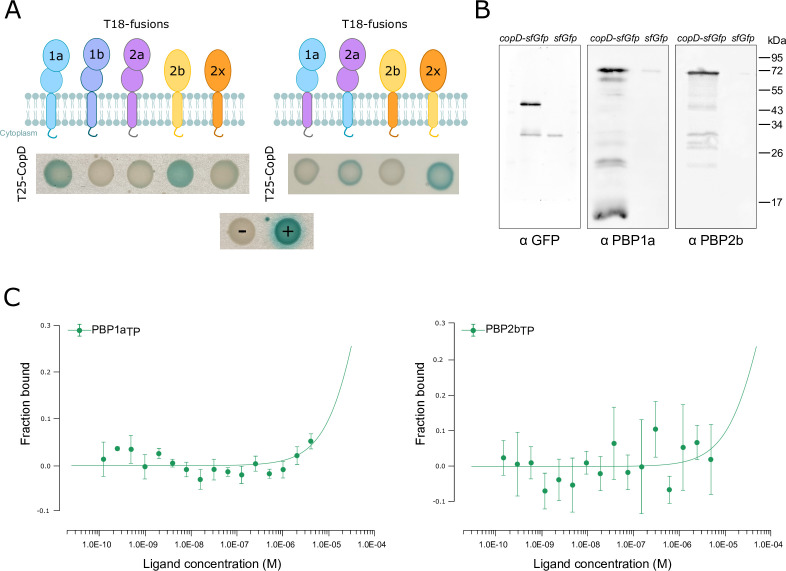
Analysis of the interaction between CopD, PBP1a and PBP2b. (**A**) Bacterial two-hybrid analyses. Plasmids, expressing the T25 fragment of the adenylate cyclase protein fused to the N-terminus of CopD or the T18 fragment fused to the N-terminus of PBP1a, PBP2b, and derivatives, were constructed and the interactions between CopD and either PBP1a or PBP2b or derivatives were assessed after co-transformation of T18- and T25-constructs in *E. coli* BTH101. The blue coloration indicates positive interactions. (**B**) Immunoprecipitation of PBP1a and PBP2b with CopD-sfGFP in *copD-sfGfp* and *P_comX_-sfGfp* strains using anti-GFP antibodies. Samples were analyzed by immunoblotting using either anti-GFP (left panel) to check that the specificity of the anti-GFP immunoprecipitation, or anti-PBP1a antibodies (middle panel) or anti-PBP2b antibodies (right panel) to determine the presence of co-immunoprecipitated mkate2-PBP1a or mkate2-PBP2b, respectively. The data shown are representatives of experiments made independently in triplicate. (**C**) Affinity measurements by Microscale Thermophoresis of labeled CopD-6His binding to increasing concentrations of either PBP1a_TP_-6his (left panel) or PBP2b_TP_-6His (right panel). The fraction bound FNorm (normalized fluorescence = fluorescence after thermophoresis/initial fluorescence) is plotted as a function of ligand concentration. Measures are represented by green dots and the fitted curve by green lines. The KD is not measurable and >10^−5^ M. Experiments were made independently in triplicate.

To further investigate the potential function of CopD in cell division and morphogenesis, we hypothesized that it could affect the activity of PBPs. Supporting this hypothesis, the CopD homolog TseB of *B. subtilis* interacts with the class B PBP PBP2A (presumably equivalent to PBP2b in *S. pneumoniae*) (28). We first searched for physical interactions between CopD and aPBPs and bPBPs using a bacterial two-hybrid screen (32). Strikingly, we detected a reproducible interaction between CopD and only PBP1a and PBP2b (Fig. 3A). To confirm this observation, we determined whether these 2 PBPs are able to interact with CopD *in vivo* by co-immunoprecipitation experiments. After CopD-sfGFP capture, PBP1a and PBP2b antibodies revealed an interaction between CopD and PBP1a and PBP2b (Fig. 3B). We then sought to better characterize how CopD interacts with PBP1a and PBP2b. We overproduced the extracellular two-PEPSY domain of CopD (from Met38 to Leu162, CopD_ED_) and the two transpeptidase domains of PBP1a (from Ser266 to Asn650, PBP1a_TP_) and PBP2b (from Met39 to Asn685, PBP2b_TP_), performed Ni-NTA affinity purification (Fig. S3) and used Microscale Thermophoresis. Surprisingly, no binding of CopD to either PBP1a_TP_ or PBP2b_TP_ was detected (KD >10 µM) (Fig. 3C). This observation suggested that the interaction between CopD and the 2 PBPs was mediated by their transmembrane domain (TM) and/or their short cytoplasmic domain (CD) (16, 12, and 12 amino acids in CopD, PBP1a, and PBP2b, respectively) (Fig. 1A). To test this hypothesis, we constructed variants replacing TM and CD of PBP1a or PBP2b with TM and CD of two other PBPs, PBP2a, or PBP2x, respectively, which did not interact with CopD (Fig. 3A). Bacterial two-hybrid assays showed that both of the PBP1a/PBP2a_TM-CD_ and PBP2b/PBP2x_TM-CD_ variants lost their ability to interact with CopD (Fig. 3A). As a control, the other two variants PBP2a/PBP1a_TM-CD_ and PBP2x/PBP2b_TM-CD_ were able to interact with CopD, although the signal was weaker than with wild-type PBP1a and PBP2b. These data thus demonstrate that the interaction between CopD and PBP1a and PBP2b is independent of their extracellular domains.

We then analyzed the localization of the three proteins to determine when CopD co-localizes with PBP1a and PBP2b during the cell cycle. We first constructed strains expressing *mkate2-PBP1a* and *mkate2-PBP2b*. The latter is expressed under the control of the native promoter at its chromosomal locus and the analysis of cell growth and cell shape confirmed that this fusion is functional, as already shown when fused to another fluorescent tag (33) (Fig. S4A and B). On the other hand, since fusions to PBP1a are not fully functional (25), we constructed a merodiploid strain carrying an ectopic *mkate2-PBP1a* fusion under the control of the zinc-inducible P_Zn_ promoter at the non-essential *bga* locus. We also confirmed that the cells grew and were shaped like WT cells (Fig. S5A and B). For both strains, we verified that mkate2-PBP1a and mkate2-PBP2b were stable and properly localized at midcell (Fig. S4C and D, S5C and D). The two strains were then transformed with *copD-sfGFP*, resulting in double-labeled strains expressing either both CopD-sfGFP and mkate2-PBP1a (*copD-sfGfp-P_Zn_mkate2-PBP1a*) or CopD-sfGFP and mkate2-PBP2b (*copD-sfGfp-mkate2-PBP2b*). As expected, the three proteins localized to the division septum and the cell equators (Fig. 2B and C). More importantly, heatmaps showed that the three proteins shared the same dynamics and co-localized throughout the cell cycle. Taken together, these observations show that CopD is able to interact with PBP1a and PBP2b, and further suggest that it could influence the function of these two PBPs at each stage of the cell cycle.

### Deletion of *copD* alters PBP1a and PBP2b dynamics

**Fig 4:**
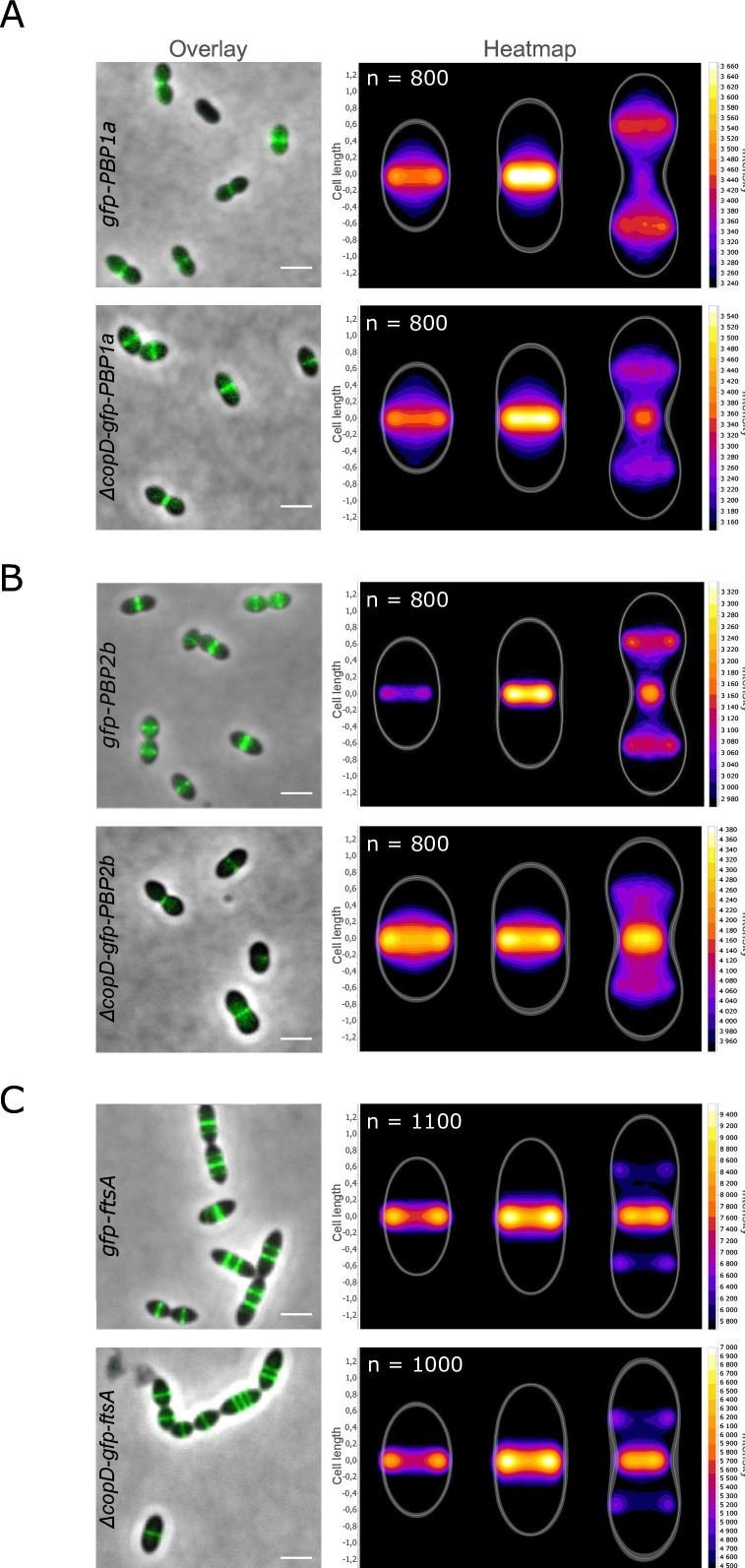
Localization of GFP-PBP1a, GFP-PBP2b, and GFP-FtsA in WT and ∆*copD* cells. (**A**) GFP-PBP1a, (**B**) GFP-PBP2b, and (**C**) GFP-FtsA. Overlays between phase-contrast and GFP images in WT and ∆*copD* cells are shown. Scale bar, 2 µm. Corresponding heatmaps representing the two-dimensional localization patterns during the cell cycle are shown on the right of overlays. The *n* values represent the number of cells analyzed in a single representative experiment. Experiments were performed in triplicate.

Since there was no interaction occurring between the extracellular domains of the three proteins, we reasoned that the mode of interaction between CopD and PBP1a or PBP2b, which requires their transmembrane and cytoplasmic domains, could modulate the localization and/or the dynamics of PBP1a and PBP2b rather than their activity. We, therefore, deleted *copD* in strains expressing *gfp-PBP1a* and *gfp-PBP2b*. The resulting strains, ∆*copD-P_Zn_gfp-PBP1a* and ∆*copD-gfp-PBP2b,* were then analyzed by microscopy. In both strains, the ability of GFP-PBP1a and GFP-PBP2b to position at mid-cell was not affected (Fig. 4A and B), indicating that CopD is not required for their localization at the division septum. Importantly, however, heatmap analysis revealed that the dynamics of both PBPs were affected. Indeed, the fluorescence signals of PBP1a and PBP2b are brighter at the division site of ∆*copD* cells at a later stage of the cell cycle than in WT cells. Simultaneously, their fluorescent signal at the daughter cell equator is less intense. As a control, and to check whether the overall divisome dynamics is also affected, we localized GFP-FtsA as a proxy for the divisome in ∆*copD* cells (Fig. 4C). No difference was detected between WT and ∆*copD* cells confirming that CopD governs the timing of localization of PBP1a and PBP2b at the division and equatorial sites.

## DISCUSSION

In this study, we show that CopD is a morphogenic protein that controls the dynamics of PBP1a and PBP2b. This control is mediated by a direct interaction between the transmembrane helices and the short cytoplasmic domains of CopD, PBP1a, and PBP2b. However, we did not detect any interaction between the extracellular domain of CopD and the TP domains of PBP1a and PBP2b. The extracellular domain of CopD is composed of 2 PEPSY domains between S47 and Q100 and Q109-E160 according to Alphafold prediction (Fig. 1A and https://alphafold.com/entry/Q8DP25). Therefore, it would be interesting to evaluate the function of the CopD PEPSY domains. PEPSY domains consist of four antiparallel β-strands and one helix, a fold that belongs to the bacterial superfamily of BLIP (β-lactamase inhibitor protein) like protein (BLIP-like proteins) (34). This family encompasses four families (DUF2874, BLIP, SmpA_OmlA, and PEPSY) of proteins with no or diverse additional domains (34). The function and thus the mode of action of these proteins is still poorly understood, but a number of them are involved in maintaining the integrity of the bacterial cell envelope (35 – 37).

PEPSY domains were initially described as intramolecular inhibitor of protease activity in the M4 family of metallopeptidases (38). However, further analyses have shown that they are present in other transmembrane proteins with different functions. For example, the three tandem PEPSY domains of YpeB are critical for the stability, localization, and activity of the lytic transglycosidase SleB required for *Bacillus anthracis* spore germination (39). Similar to the interaction between CopD and PBP1a and 2b, the PEPSY domains of YpeB don’t seem to be involved in the interaction with SleB (39, 40). Two studies report that PEPSY domain proteins participate in bacterial cell development and morphogenesis. The secreted protein SspA from *Streptomyces coelicolor* has two PEPSY domains that are required for proper spore morphology and septation (41). However, the underlying mechanism is unknown and it is only hypothesized that SspA may affect the function of some peptidoglycan hydrolases and/or PBPs. The other example is the CopD homologous protein TseB from *B. subtilis* (28). This protein was originally shown to suppress the tetracycline sensitivity of an *ezrA* mutant in *B. subtilis* (27). This property is not relevant in the pneumococcus because depletion of *ezrA* (*ezrA* is essential in *S. pneumoniae*) does not confer tetracycline sensitivity (Fig. S1). In contrast and importantly, TseB directly interacts with PBP2A, the homolog of pneumococcal PBP2b (28). In addition, *B. subtilis* cells lacking *tseB* are also wider, an observation also made with the pneumococcal ∆*copD* mutant (Fig. 1C and D). Thus, it seems that the two homologs TseB and CopD have a similar function in bacterial cell morphogenesis. Nevertheless, some properties of the two proteins differ. TseB interacts with PBP2A through its PEPSY and TP domains, respectively, according to pull-down experiments. Although this finding fits well with the inhibitory and/or stabilizing role originally proposed for PEPSY domains (39, 42), TseB is, however, not required for PBP2A activity and stability (28). The situation is different in the pneumococcus, where CopD and PBP1a and PBP2b interact through their transmembrane and cytoplasmic domains (Fig. 3). This discrepancy may reflect a real difference in the mode of action of CopD and TseB. This hypothesis is supported by the observation that TseB was not found to control the function of any aPBP in *B. subtilis* (28). In addition, CopD modulates the dynamics of PBP1a and PBP2b (Fig. 4A and B), whereas the effect of TseB on the dynamics of PBP2A remains to be evaluated.

Importantly, deletion of *PBP2A* has no detectable effect on *B. subtilis* cell shape (43), whereas deletion of *PBP1a* or depletion of *PBP2b* generates pneumococcal cells with either reduced cell size or a lentil-like shape (44, 45). Therefore, it can be suggested that TseB may regulate proteins other than PBP2A to control cell morphogenesis. Supporting this, a recent AI-assisted structural proteomics study suggests that TseB may interact with the elongasome protein MreC (46). Furthermore, the interaction between TseB and PBP2A is required for spore germination (28). Collectively, TseB would function in a different way than CopD and could also be considered as a functional homolog of SppA from *S. coelicolor* (41). This raises the question of the role of the PEPSY domains of CopD. To what extent do they contribute to the regulatory function of CopD? Are they necessary (or even detrimental) for the interaction with other partners to coordinate PBP1a and PBP2b with the divisome activity? Do they regulate the activity of other partners, and for example, some lytic enzymes required to coordinate PG primary synthesis and maturation (39)? Future work is definitely needed to address these questions.

A striking observation of this study is the presence of wider cells in absence of CopD (Fig. 1C and D). In contrast to cell elongation, how bacteria define their width is much less understood. A recent study in *B. subtilis* shows that the Rod complex moves circumferentially and produces oriented PG materials (47). This dynamic makes the sacculi more rigid and able to maintain the cell width, thus preventing cell widening. On the other hand, aPBPs do not move circumferentially and insert PG material isotropically, resulting in sacculi widening. Thus, the balance between PG assembly by the Rod system and aPBPs is critical in controlling cell width. The observed widening of cells in the absence of CopD is thus consistent with this model as CopD influences the dynamics of PBP2b, which work with RodA in the pneumococcal Rod system (48), and PBP1a, which is also part of the pneumococcal elongasome (45).

**Fig 5:**
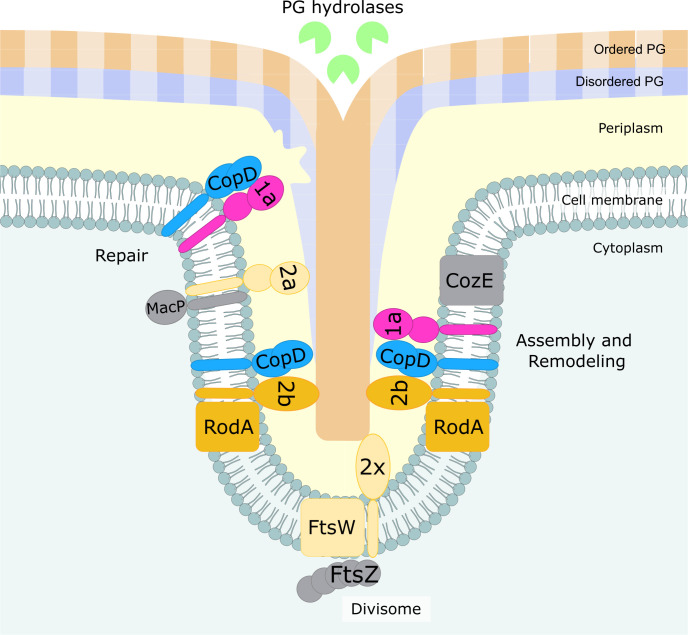
Model for CopD role in *S. pneumoniae* cell morphogenesis. CopD (light blue) modulates the dynamics of the class A PBP1a (pink) and the class B PBP2b (orange), which are both required for peripheral PG synthesis (faded salmon and blue strips). CopD could thus allow to coordinate the assembly of the primary peripheral PG with its remodeling by the RodA/PBP2b system (orange and blue) and PBP1a (pink) and hydrolases (green), respectively. This coordination is critical in controlling cell width (47). CopD could also contribute to organize the two layers PG architecture made of randomly oriented strands (blue and faded blue) facing the cytoplasm and ordered concentric rings strands (salmon and faded salmon) (49). According to the recent model of PG assembly and remodeling (21), CopD could also influence the balance of the insertion of peripheral (faded salmon and blue strips) into septal (salmon and blue strips) PG to generate a native peripheral PG mesh. Because aPBPs can function as autonomous entities, CopD could be important for PG repair and maintenance (22). Lastly, it remains to be determined if CopD cross-talks with other regulators of aPBPs like CozE and MacP (gray). The visual of this drawing was inspired by reference 50.

The larger cell width of ∆*copD* cells also fits well with the new model of pneumococcal PG assembly dynamics. Indeed, cell elongation would rely on the production of a composite PG due to the coordinated insertion of peripheral PG into the septal PG, which is continuously cleaved by hydrolases (21) (Fig. 5). The persistence of PBP1a and 2b at the division septum and the delay in their positioning at the equator (the division septum of the daughter cell) (Fig. 4AB) likely imbalance the production of peripheral PG with respect to the septal one and thus affect the composite nature of the PG resulting in cell widening. Finally, it has been demonstrated that aPBPs can function autonomously outside of the areas of active PG synthesis to repair the defects and/or damage of the primary PG (Fig. 5) (22). Therefore, CopD could be considered as a regulator that affects the function of PBP1a and PBP2b to coordinate both primary cell wall assembly and repair. This hypothesis is also consistent with the observations made by Pasquina-Lemonche and co-workers, who showed that two different PG layers with different architecture (concentric or randomly oriented) would be produced at the septum (49). Adapted to the pneumococcus, CopD could also contribute to coordinate random PG synthesis with the repair of the PG layer by controlling the spatiotemporal localization of PBP1a at the septum. On the other hand, CopD would also govern the assembly of the septal/peripheral composite PG required for cell elongation (50) (Fig. 5).

Last and most importantly, the pneumococcus encodes for several PBP regulators: CozE (also called CozEa), CozEb, MacP, and now CopD (24 – 26). While CozEa and indirectly CozEb control PBP1a, MacP targets PBP2a. Considering that, deletion of *PBP1a* reduces cell size while deletion of *PBP2a* does not (45), and that deletion of *PBP1a* and *PBP2a* is synthetically lethal (23), these two PBPs are functionally redundant but also have specific functions in PG assembly. In addition, the localization of the second pneumococcal bPBP, PBP2x, is dependent on the serine/threonine-kinase StkP (51). An indirect relationship also links PBP2b to the elongation regulator EloR/Jag/KphB since its absence suppresses the essentiality of PBP2b (52 – 54). As complicated as it may be, further work aiming at deciphering the interplay between all these regulators promises to further characterize the specific function of aPBPs and bPBPs in the assembly and maturation of the PG layer.

## MATERIALS AND METHODS

### Strains and growth conditions

*S. pneumoniae* strains were grown in C + Y or THY medium at 37°C. Cell growth was monitored automatically in JASCO V-630-BIO-spectrophotometer by optical density (OD) readings every 10 minutes at 550 nm. For growth on plate, THY agar supplemented with 3% sheep blood was used. *S. pneumoniae* mutants were obtained by transformation as previously described using the synthetic competence stimulating peptide 1 (CSP1) (55). Strains expressing genes under the control of the pComX or P_Zn_ promoter were cultured at 37°C in presence of either the inducer peptide ComS (56) or Zn 100 µM, respectively. The *E. coli* XL1-Blue strain was used for cloning, *E. coli* BL21(DE3) and BL21 star (DE3) strains for protein overexpression, and *E. coli* BTH101 strain for bacterial two hybrid experiments. Strains and plasmids used in this study are listed in Table S1 and S2, and were verified by DNA sequencing.

### Allelic replacement mutagenesis and plasmid construction

To construct *S. pneumoniae* mutants (gene deletions, ectopic gene expression GFP/mKate2 fusions), we used a two-step procedure, based on a bicistronic *kan-rpsL* cassette called Janus (57), which allows a physiological level of expression of gene derivatives at their chromosomal locus. DNA fragments encoding the extracellular domains of CopD, PBP1a, and PBP2b were obtained by PCR using chromosomal DNA from *S. pneumoniae* R800 strain as template. Full description of primers used for the construction of strains and plasmids is provided in Table S2.

### Tetracycline hypersensitivity experiments

To test tetracycline hypersensitivity, wild-type and *pComX-ezrA, ∆ezrA* strains were grown in C + Y until they reached OD_550_ = 0.1. Cell cultures were then diluted and plated on raising concentrations of tetracycline (0, 1.5, and 15 ng/mL) in presence or absence of ComS. Plates were incubated 15 hours at 37°C and CFU were counted. The relative proportion of viable CFU (CFU with tetracycline/CFU without tetracycline × 100) was then calculated.

### Microscopy techniques and image analysis

For microscopy experiments, 1 µL of exponentially growing cells was spotted onto an 1% agarose C + Y pad on a microscopy slide and covered with a coverglass. Slides were visualized with a Nikon Ti-E/B microscope fitted with an Orca-CMOS Flash4 V2 camera with a 100 Å ~ 1.45 objective. Images were collected using NIS-Elements (Nikon). Images were collected using NIS-Elements (Nikon) and were analyzed with ImageJ (http://rsb.info.nih.gov/ij/) and the MicrobeJ plugin (31). For statistical analysis, Student’s *t*-tests were performed in triplicate using the MicrobeJ.

### Bacterial two hybrid

The BACTH (Bacterial Adenylate Cyclase Two-Hybrid) system kit was used according to the manufacturer’s protocol (Euromedex) (32). Co-transformants were re-streaked on an LB agar plate supplemented with ampicillin 0.1 mg/mL, kanamycin 0.05 mg/mL, isopropyl-β-D-thiogalactopyranoside (IPTG) 0.5 mM, and X-gal 100 µg/mL. Plates were grown at room temperature and photos were taken at 24, 40, 65, and 72 hours to monitor the appearance of blue colonies. Plasmids used in this experiment are listed in Table S2.

### Co-immunoprecipitation

Cultures of *copD-sfGFP* and *PcomX-sfGFP* cells were grown at 37°C in THY medium until they reached OD_550nm_ = 0.4. After centrifugation, cell pellets were then incubated at 30°C for 30 minutes in buffer A (0.1 mM Tris-HCl, 2 mM MgCl_2_, 1M sucrose, 1:100 Protease Inhibitory Cocktail, 1 mg/mL of DNase I and RNase A), centrifuged again and then incubated in buffer B (0.1 mM Tris-HCl, 1 mM EDTA, 1% [vol/vol] Triton X-100, 1:100 Protease Inhibitory Cocktail, 1 mg/mL of DNase I and RNase A) at room temperature for 15 minutes. After centrifugation, the supernatant was incubated with the GFP-TRAP resin suspension according to the manufacturer’s instructions (Chromotech). Protein-bound GFP-TRAP resin was eluted with Laemmli buffer at 95°C for 10 minutes and analyzed by SDS-PAGE and Western blot with either anti-GFP antibodies, or anti-PBP1a or anti-PBP2a antibodies.

### Preparation of *S. pneumoniae* crude extracts and immunoblot analysis

Cultures of *S. pneumoniae* were grown in C + Y, pelleted and resuspended in Tris-HCl 10 mM pH 8, EDTA 1 mM. After cell disruption by sonication, crude extracts were analyzed by SDS-PAGE and electrotransferred onto an immobilon-P membrane (Millipore). Primary antibodies were used at 1:5,000 (anti-GFP, Amsbio), 1:20,000 (anti-PBP1a [58]), 1:20.000 (anti-PBP2b [58]), 1:10,000 (anti-Spr1400-Extra, Covalab) in TBST-BSA 1% and 1:250,000 (anti-Enolase [(59]) in TBST-BSA 5%. The goat anti-rabbit secondary antibody HRP conjugate (Biorad) was used at 1: 5,000.

### Protein purification

Recombinant plasmids overproducing CopD_ED_ and PBP2b_TP_ were transformed into the BL21(DE3) *E. coli* strain, whereas plasmids overproducing PBP1a_TP_ were transformed into the BL21 star (DE3) *E. coli* strain. The strains were grown in Luria Bertani (LB) broth at 37°C until they reached OD_600nm_ = 0.5 and then induced with 0.5 mM (IPTG) for 3 hours at 37°C. Bacterial pellets were then resuspended in buffer A (Tris-HCl 50 mM pH 7.5, NaCl 200 mM, glycerol 10%, DTT 1 mM) supplemented with 10 mM imidazole, 1 µg/mL lysozyme, 6 µg/mL DNAse/RNAse and 1× protein inhibitor Roche. After sonication and centrifugation 30 minutes at 30,000 × *g*, soluble proteins were incubated with a Ni-NTA agarose resin (Qiagen) for 30 minutes, washed with buffer A supplemented with 20 mM imidazole, and then eluted in buffer A supplemented with 150 mM imidazole (300 mM for PBP1a_TP_).

PBP2b_TP_ was then digested in dialysis buffer (HEPES 20 mM pH 7.5, NaCl 100 mM, DTT 1 mM, MgCl_2_ 1 mM, glycerol 10%) overnight in presence of TEV in a 1:40 (wt:wt) ratio. The dialyzed sample was then gel filtrated using a S75 10/300 Increase column (Cytiva) and buffer B (HEPES 20 mM pH 7.5, NaCl 100 mM, MgCl_2_ 1 mM, glycerol 10%). CopD_EC_ was dialyzed in buffer B without any TEV cleavage. Extra steps were needed to purify the TP domain of PBP1a. The extracellular domain of PBP1a was digested by trypsin (ratio 1:200[wt/wt]) in Tris-HCl 25 mM pH 8 for 30 minutes at 37°C to isolate the TP domain, as previously described (60). The TP domain was then purified by gel filtration as for PBP2b_TP_.

### Thermophoresis experiments

The Monolith NT.115 instrument was used to perform thermophoresis experiments. CopD_ED_ was labeled using the Protein labeling kit red NHS 2nd generation (Nanotemper) according to manufacturer’s instructions. For every experiment, the following settings were used: MST power = 40%; NanoRed laser, excitation power = 40%. All measurements were carried out in 20 mM HEPES pH 7.5, 300 mM NaCl, 10% glycerol, 0.1% Tween 20 detergent using standard capillaries. 6-his labeled CopD_ED_ was tested at the concentration at 168 nM with ranging concentration of PBP2b_TP_ and PBP1a_TP_ of 5.2 µM to 0.159 nM and 4.5 µM to 0.137 nM, respectively. Experiments were performed at 25°C.

## SUPPLEMENTAL MATERIAL

Supplemental Material mbio.01411-23-s0001.pdf Figures S1 to S5 and Tables S1 and S2.

ASM does not own the copyrights to Supplemental Material that may be linked to, or accessed through, an article. The authors have granted ASM a non-exclusive, world-wide license to publish the Supplemental Material files. Please contact the corresponding author directly for reuse.
