# Supplementary material for: The morphogenic protein CopD controls the spatio-temporal dynamics of PBP1a and PBP2b in Streptococcus pneumoniae
Source: mBio. 2023 Sep 20;14(5):e01411-23. doi: 10.1128/mbio.01411-23 (PMC10653890; doi:10.1128/mbio.01411-23)
Supplement: Supplemental Material — Figures S1 to S5 and Tables S1 and S2. [file mbio.01411-23-s0001.pdf]

SUPPLEMENTAL MATERIAL

The morphogenic protein CopD controls the spatio-temporal dynamics of PBP1a and PBP2b in *Streptococcus pneumoniae*

Cassandra Lenoir[1], Anaïs Pelletier[1], Sylvie Manuse[1], Hugo Millat[1], Adrien Ducret[1], Anne Galinier[2], Thierry Doan[3] and Christophe Grangeasse[1, #]

[1] Molecular Microbiology and Structural Biochemistry, UMR 5086, Université de Lyon, CNRS, Lyon, France

[2] Laboratoire de Biochimie Bactérienne, UMR 7283, Aix-Marseille Université, CNRS, Marseille, France.

[3] Laboratoire d'Ingénierie des Systèmes Macromoléculaires, UMR 7255, Aix-Marseille Université, Marseille, France.

Anaïs Pelletier and Cassandra Lenoir contributed equally to this work and are co-first authors.

# Correspondence should be addressed to Christophe Grangeasse christophe.grangeasse@ibcp.fr

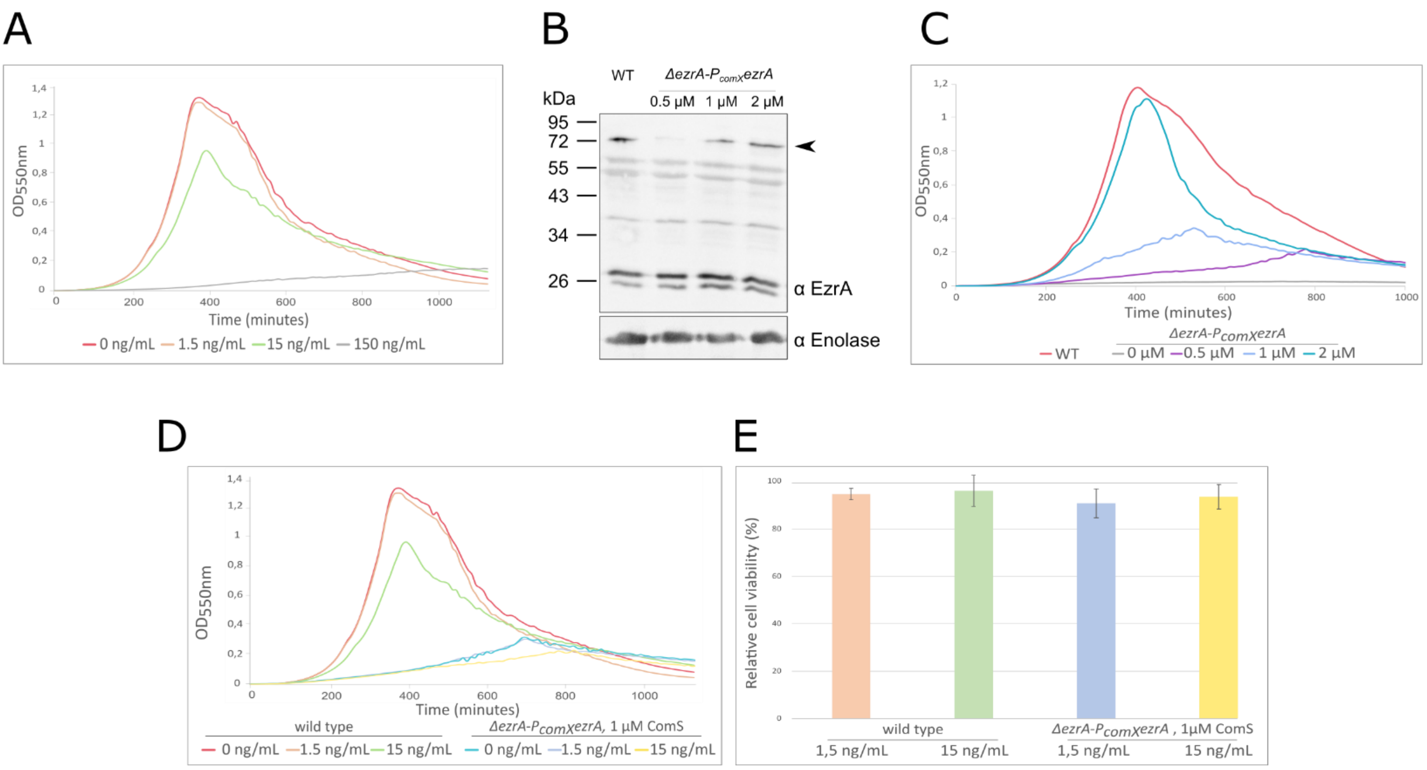

Figure S1: Effect of tetracycline and/or *ezrA*-depletion on cell growth and viability. (**A**) WT cells were grown in C+Y medium at 37°C in the presence of increasing concentration of tetracycline. The OD_{550} was read automatically every 10 min. (**B**) Western immunoblot of whole-cell lysates from Δ*ezrA*-*P*$_{comX}$-*ezrA* cells, grown to exponential phase in the presence (0.5, 1 or 2 µM) of the ComS inducer, were probed with specific anti-EzrA (α EzrA) antibody. To estimate the relative quantity of proteins in crude extract and to compare the different lanes, we used enolase (Spr1036) as an internal standard. The enolase was detected using specific antibodies (α Enolase) and is presented in the lower part of the Figure. EzrA is indicated with a black arrow. (**C**) Δ*ezrA*-*P*$_{comX}$-*ezrA* cells were grown in C+Y medium at 37°C in the absence or presence of decreasing concentration of ComS from 2 to 0 µM. The OD_{550} was read automatically every 10 min. (**D**) Growth curves of WT and Δ*ezrA*-*P*$_{comX}$-*ezrA* strains (+ 1 µM ComS) in presence of 1.5 ng/ml or 15 ng/ml of tetracycline. (**E**) Relative cell viability of Δ*ezrA*-*P*$_{comX}$-*ezrA* (+ 1 µM ComS) compared to WT cells in the presence of 1.5 ng/ml or 15 ng/ml of tetracycline. Bar chart represents the mean ± SEM.

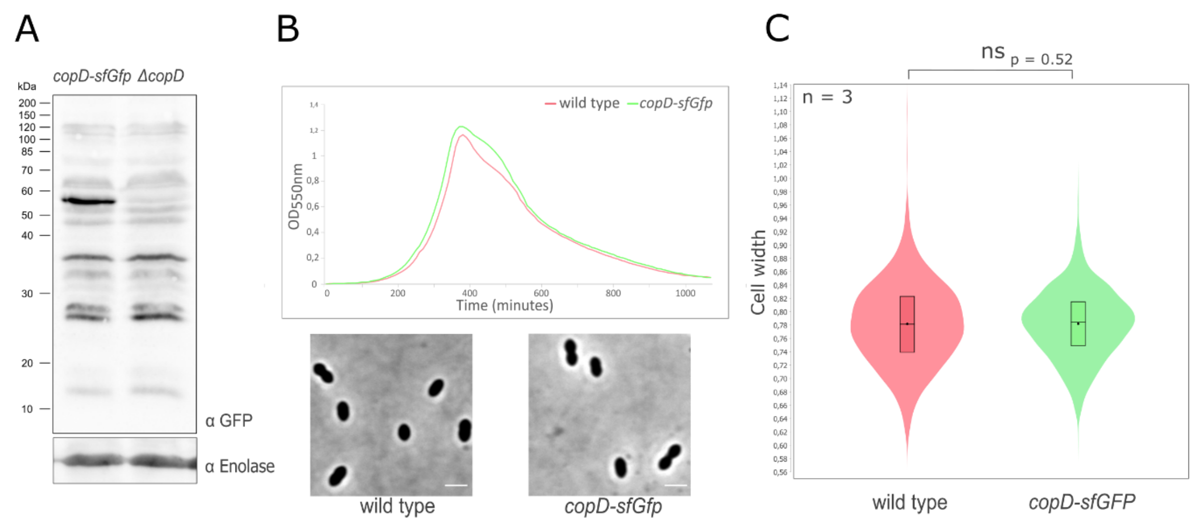

Figure S2: Expression and functionality of the CopD-sfGFP fusion. (**A**) The Western immunoblot was probed with specific anti-GFP antibodies (α GFP) to determine *copD* expression in *copD-sfGfp* cells. To estimate the relative quantity of proteins in crude extract and to compare the different lanes, we used enolase (Spr1036) as an internal standard. The enolase was detected using specific antibodies (α Enolase) and is presented in the lower part of the Figure. (**B**) Growth of WT and *copD-sfGfp* strains. Strains were grown in C+Y medium at 37°C in a spectrophotometer. The OD_{550} was read automatically every 10 min. Representative phase contrast microscopy images of WT and *copD-sfGfp* cells are shown below the growth curves. Scale bar, 2 µm. (**C**) Corresponding violin plot show the distribution of the cell width as determined using MicrobeJ (56). The distribution of the cell width is shown in red for the WT strain and in green for the *copD-sfGfp* strain. The box indicates the 25th to the 75th percentile, and the whiskers indicate the minimum and the maximum values. The mean and the median are indicated with a dot and a line in the box, respectively. Statistical comparison was done using t-test. n=3 indicates the number of independent experiments with a total of 8000 cells analyzed.

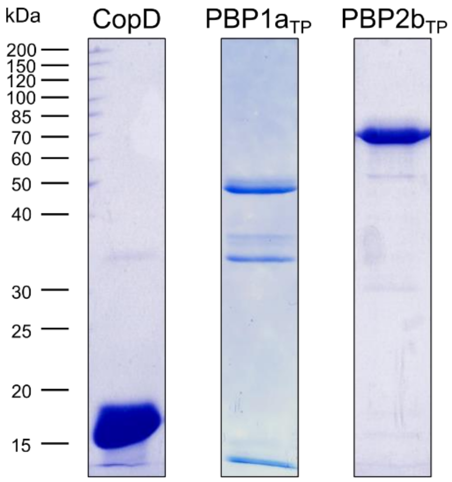

Figure S3: Purification of CopD$_{ED}$, PBP1A$_{TP}$ and PBP2b$_{TP}$. Proteins were overproduced in *E. coli* BL21 as 6his-tagged fusion proteins. After purification using a Ni-NTA resin, purified proteins were analyzed by SDS-PAGE.

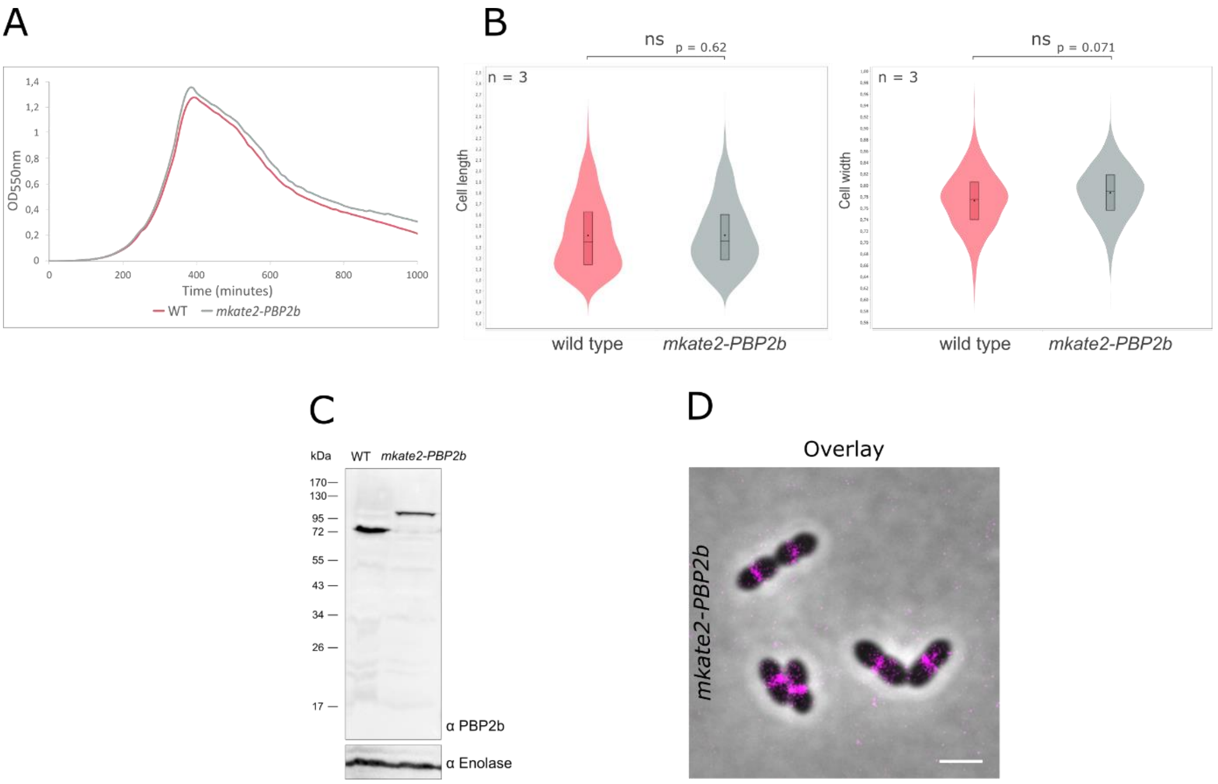

Figure S4: Validation of the *mkate2-PBP2b* fusion. (**A**) Growth curves of WT and *mkate2-PBP2b* cells in C+Y medium at 37°C. The OD_{550} was read automatically every 10 min. (**B**) Violin plot showing the distribution of the cell length (left panel) and cell width (right panel) for WT (red) and *mkate2-PBP2b* (grey) strains as determined using MicrobeJ (56). The box indicates the 25th to the 75th percentile, and the whiskers indicate the minimum and the maximum values. The mean and the median are indicated with a dot and a line in the box, respectively. Statistical comparison was done using t-test. n=3 indicates the number of independent experiments with a total of 10 000 cells analyzed. (**C**) Expression of *mkate2-PBP2b*. mkate2-PBP2b represents the only source of PBP2b from its endogenous chromosomal locus. After SDS-PAGE and electro-blotting, the membrane was probed with anti-PBP2b antibody. As control, WT cells were also analyzed. (**D**) Overlays between phase-contrast and mKate2 images of *mkate2-PBP2b* cells. Scale bar, 2 µm.

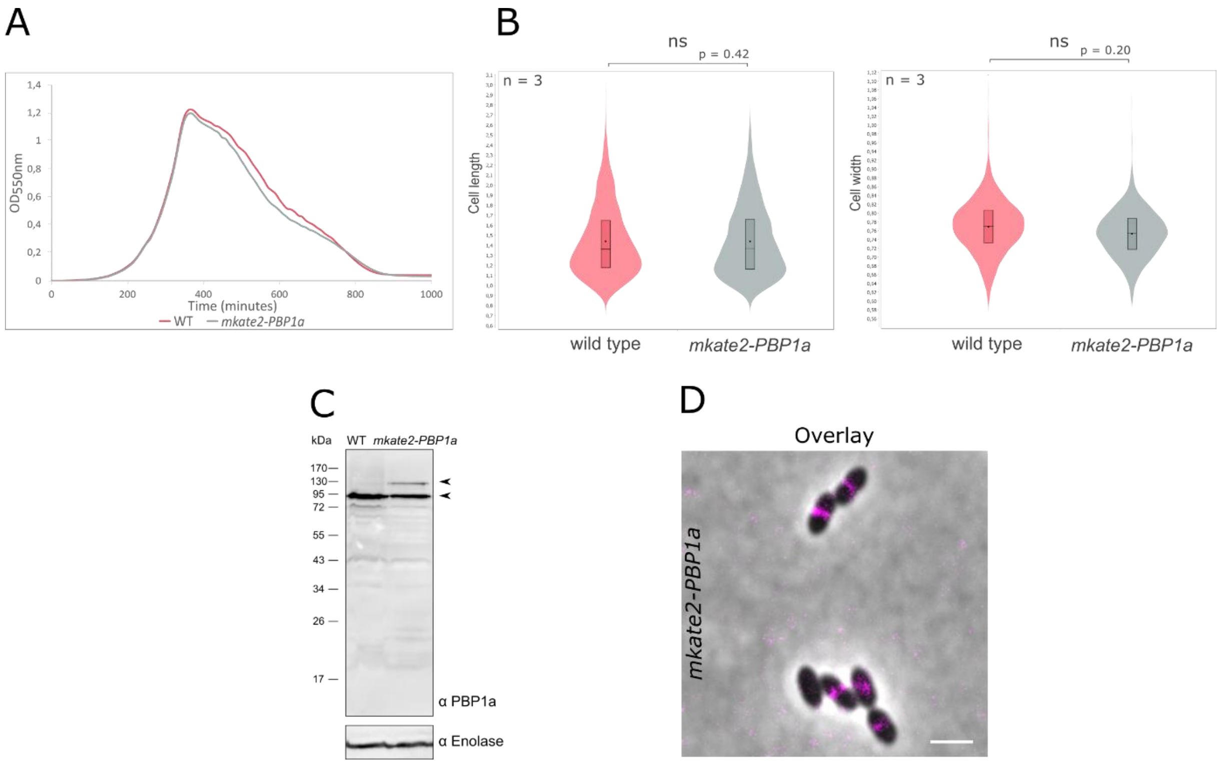

Figure S5: Validation of the *mkate2-PBP1a* fusion. (**A**) Growth curves of WT and P_{Zn}-*mkate2-PBP1a* cells in C+Y medium at 37°C. The OD_{550} was read automatically every 10 min. (**B**) Violin plot showing the distribution of the cell length (left panel) and cell width (right panel) for WT (red) and *mkate2-PBP1a* (grey) strains as determined using MicrobeJ (56). The box indicates the 25th to the 75th percentile, and the whiskers indicate the minimum and the maximum values. The mean and the median are indicated with a dot and a line in the box, respectively. Statistical comparison was done using t-test. n=3 indicates the number of independent experiments with a total of 10 000 cells analyzed. (**C**) Expression of *mkate2-PBP1a*. The merodiploid strain P_{Zn}-*mkate2-PBP1a* expresses the chromosomal *PBP1a* and the ectopic *mkate2-PBP1a* fusion under the control of the zinc-inducible P_{zn} promoter. The two forms are indicated by arrows. After SDS-PAGE and electro-blotting, the membrane was probed with anti-PBP1a antibody. As control, WT cells were also analyzed. (**D**) Overlays between phase-contrast and mkate2 images of P_{Zn}-*mkate2-PBP1a* cells. Scale bar, 2 µm.

Table S1 - Strains and plasmids used in this study

Strain	Genotype and description	Reference	Primers (Table S2)
S. pneumoniae strains			
R800	*S. pneumoniae* R6 derivative	Gift from J.-P.Claverys, Toulouse, France	
Wild type	*R800 rpsL1*	Gift from J.-P.Claverys, Toulouse, France	
copD ::Janus	*R800 rpsL1, copD::kan-rpsL; Kan^R*	This study	1, 2, 3, 4, 5, 6
ΔcopD	*R800 rpsL1, ΔcopD; Str^R*	This study	1, 2, 7, 8
copD-ΔPepSY	*R800 rpsL1, copD-ΔPepSY(A57-L162) ; Str^R*	This study	1, 2, 9, 10
copD-sfGfp	*R800 rpsL1, copD-sfGfp; Str^R*	This study	1, 2, 10, 11, 12, 13
P_{comX}-copD	*R800 rpsL1, ΔIS1167::P1::P_{comR}-comR, cpsN-O::P_{comX}-copD; Str^R*	This study	14, 15, 16, 17, 18, 19
copD ::Janus-P_{comX}-copD	*R800 rpsL1, ΔIS1167::P1::P_{comR}-comR, cpsN-O::P_{comX}-copD, copD::kan-rpsl; Kan^R*	This study	1, 2
ΔcopD-P_{comX}-copD,	*R800 rpsL1, ΔIS1167::P1::P_{comR}-comR, cpsN-O::P_{comX}-copD, ΔcopD; Str^R*	This study	1, 2
P_{comX}-sfGfp	*R800 rpsL1, ΔIS1167::P1::P_{comR}-comR, cpsN-O::P_{comX}-sfGfp; Str^R*	This study	14, 15, 16, 17, 20, 21
gfp-PBP2b	*R800 rpsL1, gfp-PBP2b; Str^R*	This study	22, 23, 24, 25, 26
copD::Janus-gfp-PBP2b	*R800 rpsL1, gfp-PBP2b, copD::kan-rpsl; Kan^R*	This study	1, 2
ΔcopD-gfp-PBP2b	*R800 rpsL1, gfp-PBP2b, ΔcopD; Str^R*	This study	1, 2
P_{Zn}-gfp-PBP1a	*R800 rpsL1, bgaA::P_{Zn}-gfp-PBP1a; Str^R Tet^R*	Gift from C. Morlot	
copD ::Janus-P_{Zn}-gfp-PBP1a	*R800 rpsL1, bgaA::P_{Zn}-gfp-PBP1a, copD::kan-rpsl; Kan^R Tet^R*	This study	1, 2
ΔcopD-P_{Zn}-gfp-PBP1a	*R800 rpsL1, bgaA::P_{Zn}-gfp-PBP1a, ΔcopD; Str^R Tet^R*	This study	1, 2
copD::Janus-gfp-PBP2b	*R800 rpsL1, gfp-PBP2b, copD ::kan-rpsl; Kan^R*	This study	1, 2
gfp-ftsA	*R800 rpsL1, gfp-ftsA; Str^R*	1	
copD-sfGfp-gfp-PBP2b	*R800 rpsL1, gfp-PBP2b, copD-sfGfp; Str^R*	This study	1, 2
P_{Zn}-mkate2-PBP1a	*R800 rpsL1, P_{Zn}-mkate2-PBP1a, Str^R Tet^R*	Gift from C. Morlot	
copD::Janus-P_{Zn}-mkate2-PBP1a	*R800 rpsL1, P_{Zn}-mkate2-PBP1a, copD::kan-rpsl, Kan^R Tet^R*	This study	1, 2
copD-sfgfp-P_{Zn}-mkate2-PBP1a	*R800 rpsL1, P_{Zn}-mkate2-PBP1a, copD-sfgfp, Str^R Tet^R*	This study	1, 2

mkate2-PBP2b	*R800 rpsL1, mkate2-PBP2b, Str^R*	This study	22, 23, 25, 27, 28
copD::Janus-mkate2-PBP2b	*R800 rpsL1, mkate2-PBP2b, copD::kan-rpsl, Kan^R*	This study	1, 2
copD-sfGfp-mkate2-PBP2b	*R800 rpsL1, mkate2-PBP2b, copD-sfGfp, Str^R*	This study	1, 2
P_{comX}-ezrA	*R800 rpsL1, ΔIS1167::P1::P_{comR}-comR, cpsN-O::P_{comX}-ezrA, Str^R*	This study	14, 15, 16, 17, 29, 30
ezrA-Janus	*R800 rpsL1, ezrA-kan-rpsL; Kan^R*	This study	3, 4, 31, 32, 33, 34
ezrA-Janus-P_{comX}-ezrA	*R800 rpsL1, ΔIS1167::P1::P_{comR}-comR, cpsN-O::P_{comX}-ezrA, ezrA-kan-rpsl ; Kan^R*	This study	31, 32
ΔezrA-P_{comX}-ezrA	*R800 rpsL1, ΔIS1167::P1::P_{comR}-comR, cpsN-O::P_{comX}-ezrA, ΔezrA ; Str^R*	This study	31, 32, 35, 36
E. coli strains			
XL1-Blue	*supE44 hsdR17 recA1 endA1 gyrA46 thi relA1 lac-F′[proAB + $lacI^q$lacZΔM15 Tn10 (Tc^R)]*	2	
BTH101	*F-, cya-99, araD139, galE15, galK16, rpsL1 (Str^R), hsdR2, mcrA1, mcrB1*	Euromedex	
BL21 (DE3)	*F-, ompT gal dcm lon $hsdS_B$ (r_B-m_B-) λ(DE3 [lacI lacUV5-T7 gene 1 ind1 sam7 nin5])*	3	
BL21 star (DE3)	*F^- ompT gal dcm lon $hsdS_B$($r_B^-m_B^-$) λ(DE3 [lacI lacUV5-T7p07 ind1 sam7 nin5]) $[malB^+]_{K-12}$(λ^S)*	Invitrogen	
Plasmids			
pETPhos-copD (extra)	*pETPhos derivative, encoding CopD, from Met38 to Leu162, Amp^R*	This study	37, 38
pETPhos-PBP2b (extra)	*pETPhos derivative, encoding PBP2b, from Met39 to Asn685, Amp^R*	This study	39, 40
pETPhos-PBP1a (extra)	*pETPhos derivative, encoding PBP1a, from Ser37 to Pro719, Amp^R*	This study	41, 42
pKT25	*P_{lac}-T25- kan pACYC ori*	4	
pUT18C	*P_{lac}-T18-, bla*	4	
pKT25-zip	*P_{lac}-T25-leucine zipper region from yeast GCN4. kan pACYC ori*	4	
pUT18C-zip	*P_{lac}-T18-leucine zipper region from yeast GCN4. bla*	4	
PUT18C-pbp2b	*P_{lac}-T18-PBP2b bla pUC ori*	5	
PUT18C-pbp2x	*P_{lac}-T18-PBP2x bla pUC ori*	This study	43, 44
PUT18C-PBP2x/PBP2bTM-CD	*P_{lac}-T18-PBP2b(Met1-Tyr38)-PBP2x(Thr52-Asp750) bla pUC ori*	This study	45, 46, 48, 49
PUT18C-PBP2b/PBP2xTM-CD	*P_{lac}-T18-PBP2x(Met1-Gly51)-PBP2b(Met39-Asn685) bla pUC ori*	This study	47, 48, 49, 50
PUT18C-pbp1a	*P_{lac}-T18-PBP1a bla pUC ori*	6	
PUT18C-pbp2a	*P_{lac}-T18-PBP2a bla pUC ori*	6	
PUT18C-PBP2a/PBP1aTM-CD	*P_{lac}-T18-PBP1a(Met1-Ser37)-PBP2a(Ser79-Arg731) bla pUC ori*	This study	51, 52, 53, 54

PUT18C-PBP1a/PBP2aTM-CD	*P_{lac}-T18-PBP2a(Met1-Lys78)-PBP1a(Lys38-Pro719) bla pUC ori*	This study	48, 55, 56, 57
pKT25-copD	*P_{lac}-T25-copD kan pACYC ori*	This study	57, 58, 59, 60

References

1 – Mercy C, Ducret A, Slager J, Lavergne J.P, Freton C, Narayanan Nagarajan S, Garcia P, Noirot-Gros M.F, Dubarry N, Nourikyan J, Veening J.W, Grangeasse C. RocS drives chromosome segregation and nucleoid occlusion in Streptococcus pneumoniae. Nature Microbiology 4: 1661-1670 (2019).

2 - Bullock, W. O., Fernandez, J. M. & short, J. M. A high efficiency plasmid transforming *recA Escherichia coli* strain with beta-galactosidase selection. Biotechniques 5, 376 (1987).

3 - Studier, F. W. & Moffatt, B. A. Use of bacteriophage T7 RNA polymerase to direct selective high-level expression of cloned genes. J Mol Biol189, 113-130 (1986).

4 - Karimova G, Ullmann A, Ladant D. Protein-Protein Interaction Between Bacillus stearothermophilus Tyrosyl-tRNA Synthetase Subdomains Revealed by a Bacterial Two-Hybrid System. *J Mol Microbiol Biotechnol* 3(1):73–82 (2001).

5 - Straume, Stamsås, Berg, Salehian, Håvarstein. Identification of pneumococcal proteins that are functionally linked to penicillin-binding protein 2b (PBP2b). Molecular Microbiology (2017) 103(1), 99–116 (2016).

6 - Fenton AK, Mortaji LE, Lau DTC, Rudner DZ, Bernhardt TG. CozE is a member of the MreCD complex that directs cell elongation in *Streptococcus pneumoniae*. *Nature Microbiology* 2(0):1–9 (2016).

Table S2 - List of primers

Number	Name	Sequence 5’-3’
1	Upstream of *copD* (-635) (+)	CACTGTACCAGGGATAGAGAAG
2	Downstream of *copD* (+1134) (-)	CACCATTGATGACCACTGTTTGC
3	Janus cassette (+)	CCGTTTGATTTTTAATGGATAATG
4	Janus cassette (-)	AGAGACCTGGGCCCCTTTCC
5	Janus / Upstream of *copD* (-1) (-)	CATTATCCATTAAAAATCAAACGGCCCTAGACCATACTGCCACAG
6	Janus / Downstream of *copD* (+488) (+)	GGAAAGGGGCCCAGGTCTCTGGTCAATAAGGAGGGCCTATG
7	Upstream of *copD* (-1) (-)	CCCTAGACCATACTGCCACAG
8	Downstream of *copD* (+411) (+)	CTGTGGCAGTATGGTCTAGGGGGTCAATAAGGAGGGCCTATG
9	*copD-ΔPepSY* (+)	AACTATCCAACCGTGTT
10	*copD-ΔPepSY* (-)	ACACGGTTGGATAGTTTCAATACTGCTGAGCT
11	Linker / *copD* (+429) (-)	CTGCAGGAACTCGATGTCTAGTTTTAGGCCCTCCTTATTGACC

12	*sfGfp* (-)	TTATTTGTAGAGCTCATCCATGCCG
13	*copD / sfGfp* (+)	CGGCATGGATGAGCTCTACAAATAAGG TCAATAAGGAGGGCCTATG
14	*cpsN* (+308) (+)	CATCGGAACCTATACTCTTTTAG
15	*cpsO* (+800) (-)	ATAACAAATCCAGTAGCTTTGG
16	P_{comX} (0) (+)	TGAACCTCCAATAATAAATATAAAT
17	*cpsN/0* (+1106) (+)	TTTCTAATATGTAACTCTTCCCAAT
18	*cpsN / copD* (-)	ATTGGGAAGAGTTACATATTAGAAATCA TAGGCCCTCCTTATTGAC
19	P_{comX} / *copD* (+)	ATTTATATTTATTATTGGAGGTTCAATGA AAAAAAGACAAAACAAAGCAAAAAATA ATC
20	P_{comX} / *sfGfp* (+)	TATTTATTATTGGAGGTTCAATGTCTAAA GGTGAAGAGTT
21	*cpsN / sfGfp* (-)	GAAGAGTTACATATTAGAAATTATTTGT ACAATTCATCCA
22	Upstream of *PBP2b* (-658) (+)	GCCGTCTTGGTCTGTCAAAGCTTC
23	Downstream of *PBP2b* (+2725) (-)	CACTATACAAAAAGAGGCCGATAGG
24	*gfp* / linker (-)	TCCGGATCCCTCGAGTTTATACAATTCAT CCATACCATGTG
25	Linker / *PBP2b* (+)	CTCGAGGGATCCGGAATGAGACTGATT TGTATGA
26	Upstream of *PBP2b / gfp* (+)	AGATTTTAAGTTAGAAATGATTTCTAAA GGTG
27	*rfp* / linker (-)	TCCGGATCCCTCGAGCAATTTACTAGGC AAAT
28	Upstream of *PBP2b / rfp* (+)	AGATTTTAAGTTAGAAATGAAACATCTT ACCG
29	P_{comX} / *ezrA* (+)	ATTTATATTTATTATTGGAGGTTCAATGT CTAATGGACAACTAATTTATTTAATGG
30	*cpsN / ezrA* (-)	ATTGGGAAGAGTTACATATTAGAAATTA AAAACGAATCGTTTCACGTGTTTTC
31	Upstream of *ezrA* (-587) (+)	CAGCCATGGGAACAGGATTTG
32	Downstream of *ezrA* (+2338) (-)	TTTATGATATTTAGCTGTACGAGAGTC
33	Upstream of Janus / *ezrA* (-)	CATTATCCATTAAAAATCAAACGGTTAA AAACGAATCGTTTCACGTGTTTTC
34	Dowtream of *ezrA* / Janus (+)	GGAAAGGGGCCCAGGTCTCTTAAAAGA AAAAGATTTTATTGTGTGAGG
35	Downstream of *erzA* (+)	CGATTGTTTCAAGTAAAAAAGGAGTTTG ATTAAAAGAAAAAGATTTTATTGTGTGA GGAGC
36	Upstream of *ezrA* (-)	GCTCCTCACACAATAAAATCTTTTTCTTT TAATCAAACTCCTTTTTTACTTGAAACAA TCG
37	Extracellular domain of *copD* with BamHI extension (-)	TATGGATCCTCATAGGCCCTCCTTATTGA CC
38	Extracellular domain of *copD* with NdeI extension (+)	TATCATATGAAACCCTATCAAACAGCTA AAAG
39	*PBP2b* with BamHI extension (-)	TATGGATCCCTAATTCATTGGATGGTAT TTTTGATACAG

40	*PBP2b* with NdeI extension (+)	GGGAATTCCATATGCAGGTTTTGAACAAGGATTTTTACG
41	*PBP1a* with BamH1 extension (-)	TATGGATCCTTATGGTTGTGCTGGTTGAG
42	*PBP1a* with NdeI extension (+)	GGGAATTCCATATGAGCAAGGCTCCTAGCCTATCCG
43	*PBP2x* with Acc65I extension (-)	TATGGTACCCGTTAGTCTCCTAAAGTTAATGTAATTTTTTTAATG
44	*PBP2x* with XbaI extension (+)	TATTCTAGAGATGAAGTGGACAAAAAGAGTAATC
45	*PBP2b* (M1-Y38) fused to *PBP2x* (T52-D750) (+)	TTGGTCGTTTGTTGTATACTCGCTTTGGAACAGA
46	*PBP2b* (M1-Y38) fused to *PBP2x* (T52-D750) (-)	TCTGTTCCAAAGCGAGTATACAACAAACGACCAA
47	pKNT25/pUT18/pUT18C-GIBSON-XbaI (-)	CTCTAGAGTCGACCTGCAG
48	pKNT25/pUT18 (+)	GTATGTTGTGTGGAATTGTGAGCGG
49	*PBP2x* (M1-M51) fused to *PBP2b* (M39-N685) (+)	TCATTATTGGGACAGGCATGCAGGTTTTGAACAA
50	*PBP2x* (M1-M51) fused to *PBP2b* (M39-N685) (-)	TTGTTCAAAACCTGCATGCCTGTCCCAATAATGA
51	*PBP1a* (M1-S37) fused to *PBP2a* (S79-R731) (+)	TTTTCTACTACGTTAGCTCGACCAATGTCAATGA
52	*PBP1a* (M1-S37) fused to *PBP2a* (S79-R731) (-)	TCATTGACATTGGTCGAGCTAACGTAGTAGAAAA
53	pUT18C (-)	TCGGGGCTGGCTTAACTA
54	pkNT25/pUT18C/pUT18 (+)	GTACCGAGCTCGAATTCA
55	*PBP2a* (M1-L78) fused to *PBP1a* (S38-P719) (+)	TGTTTGCTGTAGCCAAGAAGGCTCCTAGCCTATC
56	*PBP2a* (M1-L78) fused to *PBP1a* (L38-P719) (-)	GATAGGCTAGGAGCCTTCTTGGCTACAGCAAACA
57	pKT25-GIBSON-XbaI (-)	CTCTAGAGTCGACCCTGCAG
58	pKT25-GIBSON-Acc65I (+)	CGGGTACCTAAGTAACTAAGAATTC
59	*copD* with XbaI extension (-)	AGGGTCGACTCTAGAGGTGAAAAAAAGACAAA
60	*copD* with Acc65I extension (-)	TATGGTACCCGTAGGCCCTCCTTATTGACCAATG
